# Immunohistochemical Expression of Stanniocalcin 2 in Colorectal Cancer: A Retrospective Egyptian Study

**DOI:** 10.30699/IJP.2021-.521799.2559

**Published:** 2021-12-15

**Authors:** Hala M. El hanbuli, Rehab S. Galal, Mohammed F. Darweesh, Mohamed H. Elmahdi

**Affiliations:** 1 Pathology Department, Faculty of Medicine, Fayoum University, Faiyum, Egypt; 2 Pathology Department, Faculty of Medicine, Cairo University, Cairo, Egypt

**Keywords:** Colorectal cancer, Immunohistochemical expression, Stanniocalcin 2 (STC2)

## Abstract

**Background & Objective:**

Colorectal cancer is the third most common cause of cancer death worldwide. Stanniocalcin 2 (STC2) is a glycoprotein hormone over-expressed in many human cancers where it regulates tumor progression and invasion. Evaluating its expression in colorectal cancer and its relation with different clinicopathological parameters can provide valuable information about its role in colorectal cancer progression and behavior.

**Methods:**

This retrospective study was conducted on tissue samples of colorectal cancer. The STC2 immunohistochemical expression was detected and evaluated in 60 cases of colorectal cancer tissue samples of formalin-fixed and paraffin-embedded blocks. Then statistical analysis was performed to assess the relationship between its expression level and several clinicopathological parameters in the studied cases.

**Results:**

Statistically significant associations were found between the high level of STC2 immunohistochemical expression and histological tumor grade (*P*<0.001), tumor depth of invasion (T stage) (*P*=0.004), lymph node metastasis (N stage) (*P*=0.001), tumor Dukes’ stage (*P*<0.001), the presence of lymphovascular invasion (*P*<0.001), and perineural invasion (*P*<0.001).

**Conclusion:**

STC2 over-expression in colorectal cancer may be associated with more aggressive tumor behavior including increased tumor invasion, higher histological grade, higher rate of nodal metastasis and increased incidence of lymphovascular and perineural invasions. These data suggest a potential role for STC2 as a predictive biomarker for tumor behavior in colorectal cancer patients.

## Introduction

Colorectal cancer (CRC) is the fourth most commonly diagnosed cancer and the 3^rd^ most common cause of cancer death worldwide (1). It is also the 2^nd^ most common cancer in females following breast cancer and the 3^rd^ most common cancer in males following prostate and lung cancers (2).

The 5-year survival rate is 90% for the localized CRC, while CRC associated with lymph nodes or distant metastasis has a survival rate ranging from 14% to 71% (3). Tumor depth of invasion (T), lymph node status (N), tumor grade, and lymphatic and venous invasion are the most important histopathological prognostic factors (4), while clinical outcomes and patient survival are improved by screening programs and new treatment protocols, including targeted therapies. The main causes of patients’ mortality are local recurrence and distant metastasis (5). Therefore, the ability to predict the risk of both tumor aggressive local invasion and tumor metastasis can change and improve the clinical management of CRC patients.

Stanniocalcin 2 is a disulfide-linked homodimeric glycoprotein hormone formed of 302 amino acids and coded by STC2 gene on chromosome 5q35.2 (6) and is normally expressed in many human tissues, including proximal and distal convoluted tubules and collecting duct in the kidney, hepatocytes near central veins, alpha cells in the pancreas, adipose tissue, small and large intestine, smooth muscles, heart, spleen, lung, placenta, skeletal muscles, and brain (6,7). 

Stanniocalcin 2 has many physiological functions, including calcium and phosphate homeostasis (8), osteoblast differentiation and mineralization (9), regulation of postnatal growth (10), angiogenesis and vascular sprouting in cases of hypoxia and ischemia (11), and protection against stress as it protects cells against apoptosis caused by insulting events such as oxidative stress, hypoxia, glucose deprivation, and endoplasmic reticulum stress through up-regulation of Cyclin-dependent kinase 2 and 4 (CDK2 and 4), down-regulation of cell cycle inhibitors p16 and p21, and activation of pAKT and pERK1/2 signaling pathway, which cause to increase cell viability and survival even after cell exposure to injurious stimuli (12)(13). 

Stanniocalcin 2 over-expression is detected in various human cancers where it helps tumor cells to adapt to the stressful conditions in tumor microenvir-onment with hypoxia and glucose deprivation, thus increasing tumor ability to invade and progress. Recent studies indicated that STC2 was overexpressed in hepatocellular carcinoma, neuroblastoma, glioblastoma, renal cell carcinoma, esophageal squamous cell cancer, prostate, gastric, lung, head and neck, and ovarian cancers (14)(15), and high expression of STC2 in all above-mentioned tumors was correlated with the increased tumor growth, migration, invasion, increased incidence of nodal and distant metastasis, decreased survival and overall poor prognosis (6)(16). STC2 overexpression has been also reported in breast cancer, but is associated with a good prognosis (14)(17). 

This study evaluated the level of STC2 immunohis-tochemical expression in 60 CRC cases and explored its association with different clinicopathological parame-ters.

## Material and Methods

**Tissue Collection**

Sixty cases of colorectal cancer were retrospect-tively collected from archived paraffin blocks from Pathology Department, Faculty of Medicine, Cairo University. All specimens were obtained through colectomy procedure during the period between January 2018 and June 2019. 

**Routine Haematoxylin and Eosin (HE) staining**

The staining was performed for the morphological analysis of the studied cases.

**Immunohistochemical** **Staining**

Tissue sections with 4 µm thickness were mounted on positively charged slides followed by routine de-paraffinization and antigen retrieval by citrate buffer (pH 6.0) in an automated water bath (Dako PT link, PT101), then, immunohistochemical staining was performed using anti-STC2 antibody (polyclonal rabbit anti-STC2 antibody IgG, 0.1 mL at 200 µg/mL purified by peptide affinity chromatography using Sulfo Link Coupling Resin, diluted at 1:50, manufactured by Thermo Fisher Scientific, U.S.A.) by an autostainer (Dako autostainer link 48) using a polymer-based detection system (Dako En Vision TM FLEX, K8000). The positive brown staining was visualized and photographed using a light microscope (BX51; Olympus Corporation, Tokyo, Japan).

**STC2 staining score**

STC2 staining score was calculated as introduced by Zhen-Hai Zhang* et al.*, (18). The product of the percentage positivity of the stained tumor cells and the staining intensity were as follow; the percentage positivity <5%, scored 0; ≥5 to <25%, scored 1; ≥25 to <50%, scored 2; ≥50 to <75%, scored 3; and ≥75%, scored 4. Staining intensity was scored as score 0: no staining; score 1: mild staining; score 2: moderate staining; and score 3: strong staining. 

The final STC2 immunostaining score ranges from 0 to 12 as follows: no expression (score of 0-3) and weak expression (score of 4-6) are considered negative STC2 expression, low-grade positive expression (**+**) (score of 7-9), and high-grade positive expression (**++**) (score of ≥10).

**Statistical Analysis**

The statistical analysis was performed using the SPSS version 16 (SPSS Inc., Chicago, IL. USA). Data were presented using descriptive statistics in the form of frequencies and percentages for the qualitative data and mean ± standard deviation (SD) for the quantitative variables. Chi-square test (X2) was used for the comparisons between STC2 expression subgroups. The level of statistical significance was set at probability P-value<0.05 (19).

## Results

Of the 60 cases of CRC included in this study, 17 patients (28.33%) had negative STC2 staining (7 no staining and 10 weak staining, and both were considered negative according to our scoring system), 32 patients (53.33%) showed mild positive staining, and 11 patients (18.33%) demonstrated intense positive staining (Figure 1). 

The association between the STC2 expression level and clinicopathological features was calculated by the Chi-squared test. Data are summarized in Table 1.

High STC2 expression in CRC tissues showed a statistically significant relation with histological tumor grade (*P*<0.001), tumor depth of invasion (T stage) (*P*=0.004), lymph node metastasis (N stage) (*P*=0.001), Dukes’ stage (*P*<0.001), lymphovascular invasion (*P*<0.001), and perineural invasion (*P*<0.001). There was no statistically significant association between the STC2 expression and other clinicopathological variables, such as gender (*P*=0.117), age (*P*=0.935), tumor location (*P*=0.249), tumor size (*P*=0.816), Gross appearance (*P*=0.139), and histological type (*P*=0.404).

**Fig 1. a:**
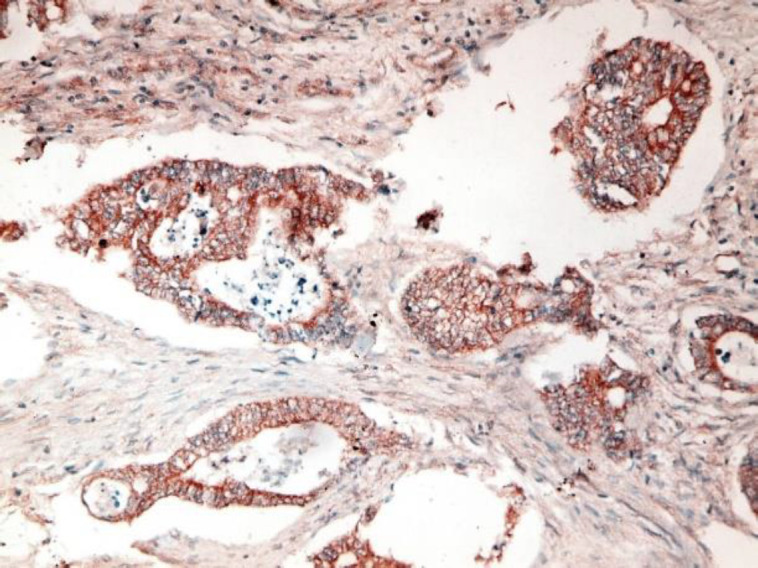
Poorly differentiated CRC with intense positive cytoplasmic STC2 staining (IHC staining X40)

**Fig 1. b:**
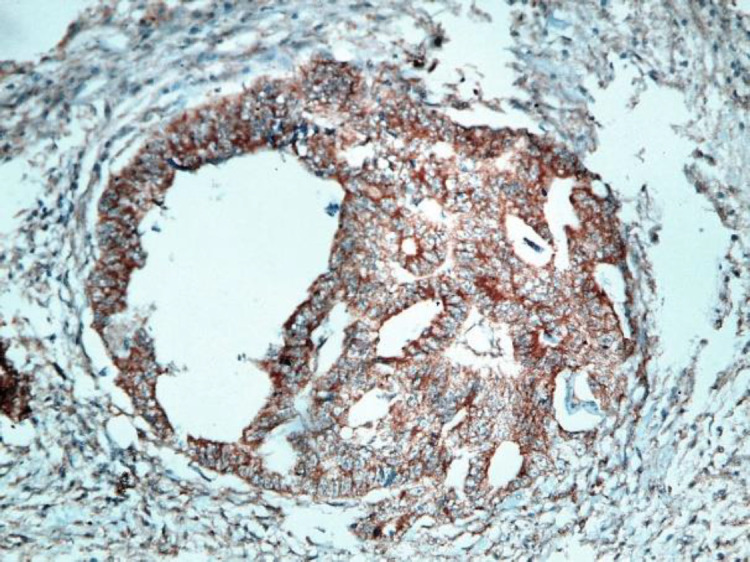
Moderately differentiated CRC with intense positive cytoplasmic STC2 staining (IHC staining X40)

**Fig 1. c:**
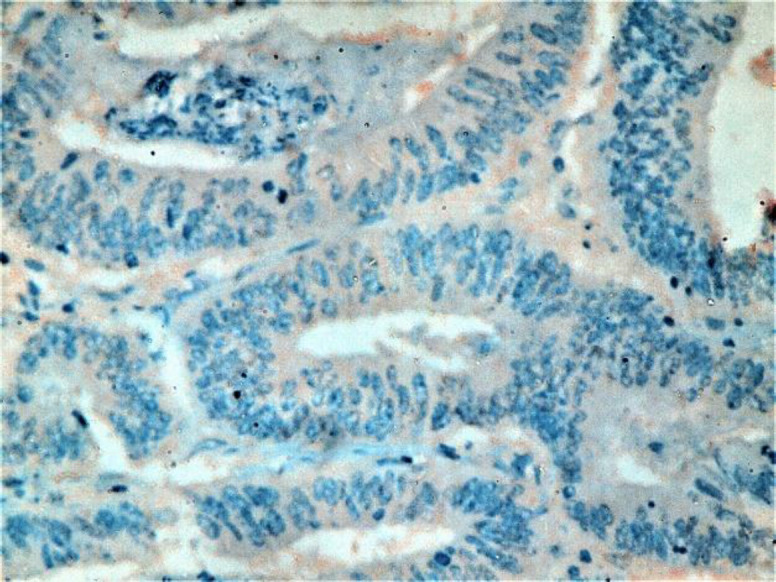
Moderately differentiated CRC with mild positive cytoplasmic STC2 staining (IHC staining X400)

**Fig 1. d:**
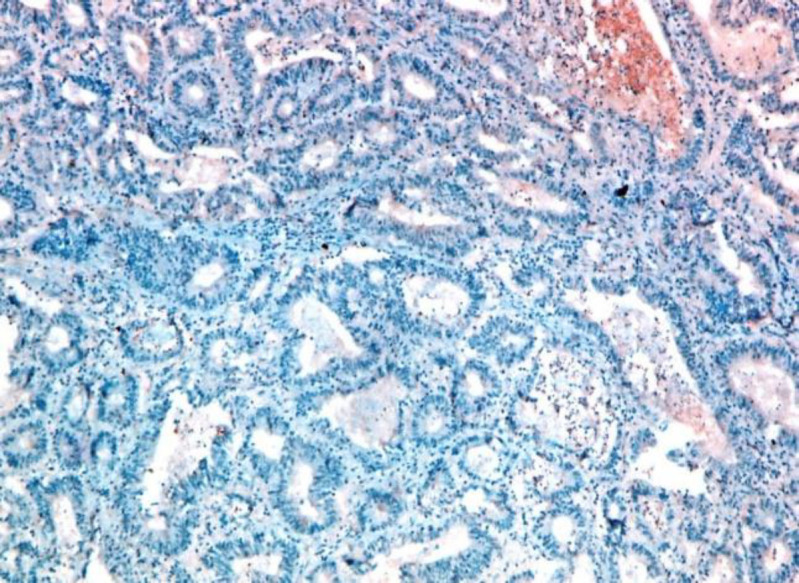
Moderately differentiated CRC with negative STC2 staining (IHC staining X20)

**Table 1:** Relation between STC2 immunohistochemical expression and clinicopathological parameters in 60 CRC patients

Clinicopathological parameter	Number (%)	STC2 expression level			P-value
		Negative	Mild positive	Intense positive	
Gender					**0.117**
Male	30 (50)	11	12	7	
Female	30 (50)	6	20	4	
Age					**0.935**
≥ 40 years	46 (76.7)	13	25	8	
< 40 years	14 (23.3)	4	7	3	
Tumor size, cm					**0.816**
≥ 5 cm	33 (55)	9	17	7	
< 5 cm	27 (45)	8	15	4	
Gross appearance					**0.139**
Fungating	26 (43.3)	4	17	5	
Ulcerating	21 (35)	10	7	4	
Infiltrating	13 (21.7)	3	8	2	
Tumor site					**0.249**
Colon	48 (80)	13	24	11	
Rectum and sigmoid	12 (20)	4	8	0	
Histological type					**0.404**
Adenocarcinoma	58 (96.7)	17	30	11	
Mucinous	2 (3.3)	0	2	0	
Tumor Grade					**<0.001**
Grade II	54 (90)	17	31	6	
Grade III	6 (10)	0	1	5	
Tumor T-Stage					**0.004**
T2	3 (5)	2	1	0	
T3	46 (76.7)	15	26	5	
T4	11 (18.3)	0	5	6	
Lymph Node Metastasis					**0.001**
N0	31 (51.7)	13	16	2	
N1 (1-3 regional L.N)	17 (28.3)	3	12	2	
N2 (≥ 4 regional L.N)	12 (20)	1	4	7	
Distant Metastasis					**----**
Mx	56 (93.3)				
M1	4 (6.7)	0	0	4	
Modified Dukes’ stage					**<0.001**
B1	1 (1.66)	1	0	0	
B2	30 (50)	12	16	2	
C1	1 (1.66)	1	0	0	
C2	24 (40)	3	16	5	
D	4 (6.66)	0	0	4	
LVI					**<0.001**
Negative	50 (83.3)	17	30	3	
Positive	10 (16.7)	0	2	8	
PNI					**<0.001**
Negative	52 (86.7)	17	31	4	
Positive	8 (13.3)	0	1	7	

Abbreviations: (LVI) Lymphovascular invasion, (PNI) Perineural invasion.

Note: Bold indicates statistically significant values (*P*<0.05).

## Discussion

In Egypt, colorectal cancer is the 7^th^ most commonly diagnosed cancer, representing 3.47% of cancers in males and 3% in females (20). It has a relatively high incidence at the age under 40 years (1.3/10^5^). While the incidence in the age groups 40-59 years, 60-69 years and > 70 years are 12.0/10^5^, 19.4/10^5^ and 21.2/10^5^**,** respectively (21). 

Only a few studies previously focused on the STC2 expression in colorectal cancer and most of these studies were experimental or focused on DNA analysis (22-27). These studies indicated that STC2 gene silencing in CRC cells inhibits cell viability, invasion and migration, while STC2 over-expression increases EMT as it activates the phosphorylation of ERK, MEK and AKT signaling pathways, increases N-cadherin, vimentin and TWIST, decreases E-cadherin and upregulates P-glycoprotein. The STC2 also regulates the growth and migration of colon cancer cells under hypoxic conditions and reduces patients’ response to certain chemotherapeutic drugs. 

To the best of our knowledge, only one recent study was performed to evaluate the immunohistochemical expression of STC2 in colon and rectal cancer and its relation to the tumor clinicopathological features by Zhang* et al.*, and concluded that STC2 is an independent prognostic factor in colorectal cancer patients and the elevated STC2 expression is strongly correlated with the presence of nodal and distant metastasis, advanced clinical stage, and worse clinical outcome (15), and the results of this study was compared to that of Zhang* et al.*, study.

In this study, STC2 was expressed in 71.7% of the 60 CRC cases (53.3% low grade positive **+**, and 18.3% high-grade positive **++**) and 28.3% of the studied cases were STC2 negative. While in Zhang* et al.*, study, negative STC2 expressions were found in 24.35% of the 115 cases included in their study and 75.6% of their cases expressed STC2 (15). 

The STC2 expression in this work was intensely positive in 13.3% of the studied cases ≥ 40 years and in 5% of cases < 40 years old with no statistically significant relation between the STC2 expression and patients’ age (*P*=0.935). Also, intensely positive STC2 expression was detected in 23.3% of males and in 13.3% of females and there was no statistical relationship between the STC2 expression and the patients’ gender (*P*=0.117). Zhang* et al.* (15) showed in their work that high expression level of STC2 was found in 47.5% of males and 42.6% of females with no statistical relationship between the STC2 expression and the patients’ gender (*P*=0.595) and also found no statistically significant relation between the STC2 expression and the patients’ age (*P*=0.932).

Our study showed that 28.6% of lesions located in the ascending colon were intensely positive for STC2, and the majority of lesions located in the transverse, descending and sigmoid colon showed mild positivity for STC2 (75%, 68.8% and 63.6% of cases, respectively). Only 1 case of rectal cancer included in this study was intensely positive for the STC2. The relation between the STC2 expression and tumor site was statistically insignificant (*P*=0.249). In addition, no significant relationship was found between the STC2 expression and the tumor’s gross appearance (*P* =0.139). Zhang* et al.* (15) also found no statistically significant relation between the STC2 expression and tumor site (*P*=0.136) but with incidence rates of 51.6% of colon cancers and 37.7% of rectal cancers showing high expression of STC2. They did not demonstrate a relationship between the STC2 and the gross appearance of CRC.

This study showed no significant relationship between the STC2 expression and tumor size (*P*=0.816) or tumor histopathological type (*P*=Z0.404). The same results regarding tumor size were found by Zhang* et al.* (15). They did not express a relation between STC2 and tumor grade, while in this study, a statistically significant relation was found (*P*<0.001) between the previously mentioned variables. 

According to Kim* et al.*, tumor T stage is one of the five independent factors which affect overall survival in CRC patients (28) and tumor size is a significant prognostic and predictive factor especially for T1 colon cancer as large horizontal tumor extension is associated with poor prognosis (29).

The results of this study indicated a statistically significant relation between the STC2 expression and T stage (*P*=0.004). But in contrast to this result, Zhang* et al.*, (15) found no significant relationship between the STC2 expression and tumor T stage (*P*=0.205). 

Lymph node metastasis (N stage) is an important prognostic factor in the patients with CRC and a higher lymph nodes ratio (the ratio of the positive lymph nodes to the total lymph nodes harvested) is predictive for both overall and disease-free survival (30).

In this study, a statistically significant relation was found between the STC2 expression and tumor N stage (*P*=0.001). Zhang* et al.* (15) also reported a statistically significant relation between the STC2 expression and nodal metastasis.

A relation between the STC2 expression and distant metastasis could not be assessed in this study as 56 out of 60 studied cases were Mx and only 4 cases were identified as being associated with M1 stage and all 4 cases expressed high grade positive STC2 staining. Zhang* et al.* (15) found a statistically significant relation between the STC2 expression and distant metastasis (*P*=0.040) with high expression of STC2 in 70.6% of cases with distant metastasis and in 40.8% of cases without distant metastasis.

With regards to Dukes’ classification in this study, high grade positive STC2 expression was found in 100% of Dukes’ D stage (all 4 cases), 20.8% of Dukes’ C2 stage, and in 6.7% of Dukes’ B2 stage. Statistically significant relation was found between the STC2 expression and tumor Dukes’ stage (*P*<0.001).

According to Cienfuegos* et al.*, perineural invasion is a major prognostic and predictive factor in colon cancer as PNI positive patients had a significantly lower disease-free survival at 5 years than patients without PNI (31) and both perineural invasion and lymphovascular invasion were associated with poorer disease-free survival and overall survival (32).

In this study, all cases with lymphovascular invasion were positive for the STC2 expression and a significant relation was found between the STC2 expression and lymphovascular invasion (*P*<0.001) and perineural invasion (*P*<0.001). Our results were different from Zhang* et al.* (15) results which stated no statistically significant relationship between the STC2 expression and lymphovascular invasion or perineural invasion.

A new emerging therapeutic role of STC2 in CRC has been expected and some recent studies aimed at testing this possibility.

A study by Miyazaki* et al.* in 2014 on immunohistochemical analysis of the STC2 expression levels in the patients with colorectal liver metastasis who underwent hepatectomy after receiving conven-tional chemotherapy with and without bevacizumab showed increased STC2 expression, which was higher in colorectal liver metastases treated with bevacizumab than patients without bevacizumab treatment. These data indicate that hypoxia-induced by anti-VEGF antibody therapy increases the STC2 expression and malignant potential of colorectal cancers and contributes to the treatment failure, which may explain the limited clinical effectiveness of anti-angiogenic agents (33).

It was recently discovered that STC2 gene silencing in the CRC cells inhibited cell viability, invasion and migration compared to the control cells. Meanwhile, E-cadherin increased, while vimentin, MMP-2 and MMP-9 decreased. These data showed that silencing STC2 treatment conferred a strong protective effect on CRC (34).

## Conclusion

The STC2 immunohistochemical expression in cases of CRC may be strongly related to some clinicopathological para-meters including histological tumor grade, tumor depth of invasion, lymph node metastasis, tumor Dukes’ stage, the presence of lymphovascular invasion, and perineural invasion. These data suggest a potential role for the STC2 as a predictive biomarker for tumor aggression and behavior in the CRC patients. This study also had some limitations, such as small sample size and the inability to test for the familial CRC and microsatellite instability to correlate with the STC2 expression.

## Ethics Approval

 The study was approved by the Institutional Medical Ethical Committee.

## Consent to Participate

Not applicable

## Conflict of Interest

The authors declare that they have no competing interests.

## Funding

The authors would like to confirm that they covered the expenses of this research work completely on their own and they were not funded by any institution in Egypt.

## Authors' Contributions

 The manuscript has been read and approved by all the authors

**Category 1: ** a) Conception and design: 1^st^ author. b) Analysis of data: All authors c) Interpretation of data: All authors** Category 2:** a) Drafting the article: All authors b) Revising it critically for important intellectual content: All authors** Category 3: **Final approval of the version to be published: All authors.
